# Neural networks for predicting etiological diagnosis of uveitis

**DOI:** 10.1038/s41433-024-03530-2

**Published:** 2024-12-20

**Authors:** Robin Jacquot, Lijuan Ren, Tao Wang, Insaf Mellahk, Antoine Duclos, Laurent Kodjikian, Yvan Jamilloux, Dinu Stanescu, Pascal Sève

**Affiliations:** 1 Department of Internal Medicine, Hôpital Universitaire de la Croix-Rousse, Hospices Civils de Lyon, University Claude Bernard-Lyon 1, Lyon, France; 2 Research on Healthcare Performance (RESHAPE), INSERM U1290, University Claude Bernard Lyon 1, Lyon, France; 3 School of Software Engineering, Chengdu University of Information Technology, Chengdu, China; 4 DISP UR4570, Jean Monnet Saint-Etienne University, INSA Lyon, Lyon 2 University, Claude Bernard-Lyon 1 University, Roanne, France; 5 Department of Ophthalmology, Hôpital Universitaire de la Croix-Rousse, Hospices civils de Lyon, Université Claude Bernard-Lyon 1, Lyon, France; 6 Department of Ophthalmology, Hôpital Universitaire de la Pitié-Salpêtrière, APHP, Paris, France

**Keywords:** Uveal diseases, Outcomes research

## Abstract

**Background/objectives:**

The large number and heterogeneity of causes of uveitis make the etiological diagnosis a complex task. The clinician must consider all the information concerning the ophthalmological and extra-ophthalmological features of the patient. Diagnostic machine learning algorithms have been developed and provide a correct diagnosis in one-half to three-quarters of cases. However, they are not integrated into daily clinical practice. The aim is to determine whether machine learning models can predict the etiological diagnosis of uveitis from clinical information.

**Methods:**

This cohort study was performed on uveitis patients with unknown etiology at first consultation. One hundred nine variables, including demographic, ophthalmic, and clinical information, associated with complementary exams were analyzed. Twenty-five causes of uveitis were included. A neural network was developed to predict the etiological diagnosis of uveitis. The performance of the model was evaluated and compared to a gold standard: etiological diagnosis established by a consensus of two uveitis experts.

**Results:**

A total of 375 patients were included in this analysis. Findings showed that the neural network type (Multilayer perceptron) (NN-MLP) presented the best prediction of the etiological diagnosis of uveitis. The NN-MLP’s most probable diagnosis matched the senior clinician diagnosis in 292 of 375 patients (77.8%, 95% CI: 77.4–78.0). It achieved 93% accuracy (95% CI: 92.8–93.1%) when considering the two most probable diagnoses. The NN-MLP performed well in diagnosing idiopathic uveitis (sensitivity of 81% and specificity of 82%). For more than three-quarters of etiologies, our NN-MLP demonstrated good diagnostic performance (sensitivity > 70% and specificity > 80%).

**Conclusion:**

Study results suggest that developing models for accurately predicting the etiological diagnosis of uveitis with undetermined etiology based on clinical information is feasible. Such NN-MLP could be used for the etiological assessments of uveitis with unknown etiology.

## Introduction

### Background

Uveitis is an intraocular inflammation with an incidence of 17–52 per 100,000 people and its prevalence ranges from 38 to 284/100,000 persons [1]. It ranks as the fifth leading cause of blindness worldwide, primarily due to complications like macular edema, ocular hypertonia, or retinal ischemia [2]. In 2021, The Standardization of Uveitis Nomenclature (SUN) developed classification criteria for uveitis [3]. The condition encompasses nearly 80 etiologies originating from various sources, such as pure ophthalmological-related diseases, infectious agents, inflammatory factors, masquerade syndromes, and drug-induced reactions [4]. The epidemiology of causative factors varies across countries, influenced by genetic factors (e.g. HLA-B27 and sarcoidosis), environmental elements (e.g. tuberculosis), and results of complementary examinations (e.g. nuclear imaging). Additionally, the diagnosis is influenced by ophthalmological (e.g. anatomic location, laterality, chronicity, associated signs) and extra-ophthalmological (e.g. clinical examination) characteristics, making the etiological diagnosis complex [5].

To date, only a few retrospective studies have focused on the most relevant diagnostic approach for assessing the underlying etiology of uveitis [6–9]. A single controlled study compared a “standardized 3-step approach” taking into account simple ophthalmological characteristics, to an “open” strategy with no significant difference [10, 11]. Although the algorithmic approach for etiological diagnosis appears promising, its implementation by inexperienced clinicians is challenging due to the vast amount of data that needs to be considered [4, 12].

### Prior work

In recent years, the development of machine learning techniques has offered an opportunity to harness knowledge from extensive datasets. Some Bayesian belief networks, multilayered rule-based expert systems, and decision trees have been explored for uveitis diagnosis with encouraging results [13–18]. These algorithms encompass demographic, ophthalmological, extra-ophthalmological, complementary examination, and treatment response factors. Reported evaluations of these diagnosis decision support systems demonstrated correct diagnosis in 54–74% of cases [19]. Integrating such algorithms into clinical management could lead to better diagnosis and potentially reduce the number of uveitis cases labeled as idiopathic. However, current computer-aided systems have limitations, including a limited number of etiologies, restricted training datasets, and some study factors not available immediately after uveitis diagnosis. Consequently, these algorithms are not widely used in daily clinical practice.

### Goal of this study

Developing a new diagnosis decision support system for the etiological assessment of the underlying cause of uveitis with unknown etiology could yield improved diagnostic performance.

## Materials and methods

### Dataset

**Fig. 1:**
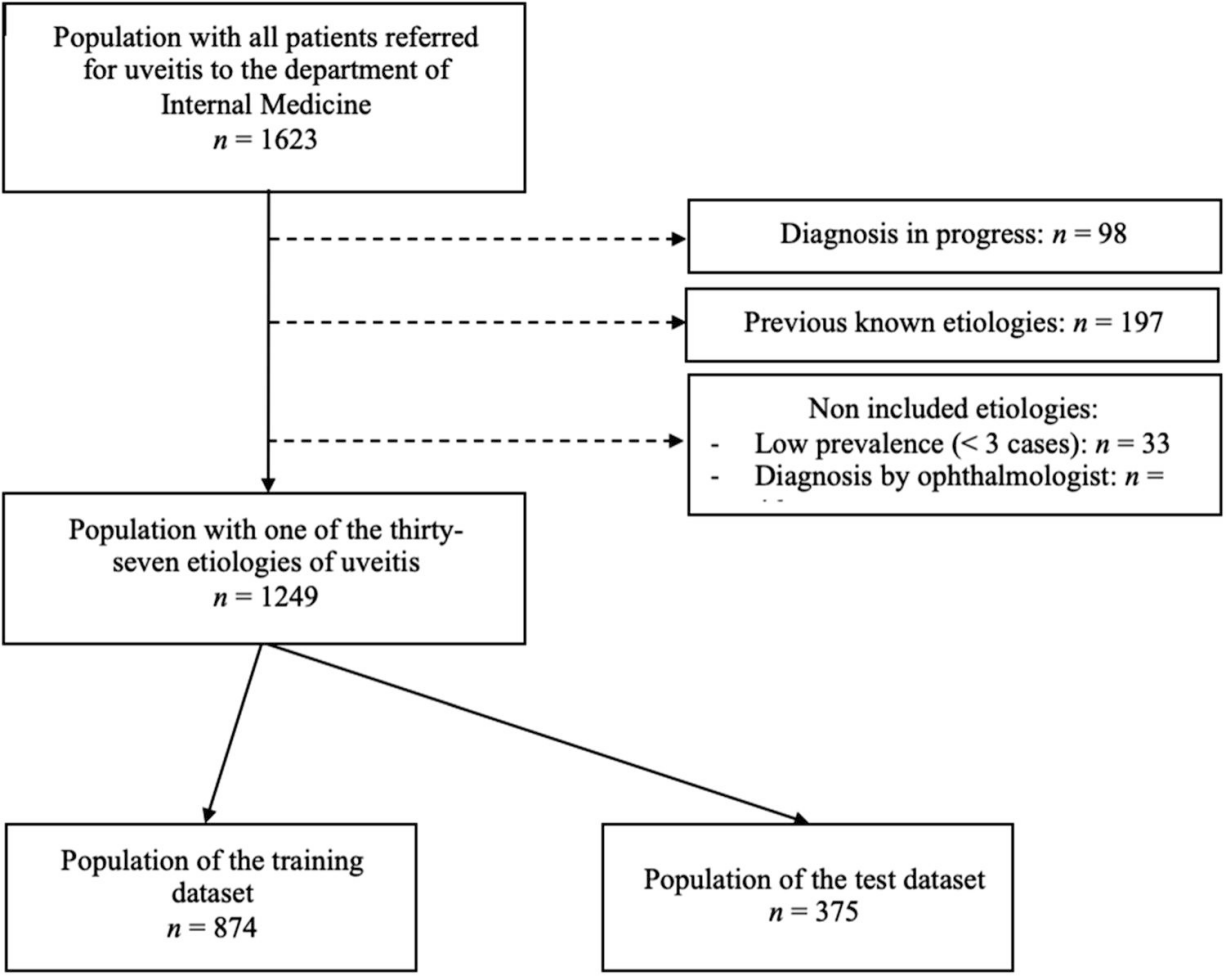
Flow chart for the training and test datasets.

The dataset was composed of incident cases of uveitis with unknown etiology after an examination by ophthalmologists. One thousand two hundred forty-nine patients are allocated randomly into training (874) and test (375) subsets (Fig. 1). Uveitis diagnosis was achieved after an ophthalmological examination. According to the anatomical classification of uveitis, diagnostic workup followed a systematic approach in line with the ULISSE screening protocol [10]. After a comprehensive assessment by an internist, 766 patients (61.3%) received an etiological diagnosis of uveitis and 483 (38.7%) remained of undetermined etiology. All patients were admitted to the department of internal medicine at the Croix-Rousse University Hospital, Lyon, between 1 January 2003 and December 2021. Patients whose etiological diagnosis was previously known at the internal medicine consultation or in progress were excluded. We removed etiologies with fewer than three cases in our cohort and/or diagnosed solely by an ophthalmologist (including only anterior uveitis requiring topical treatment).

### Definitions

The SUN was used throughout this study for the anatomic classification of uveitis [20]. Uveitis of infectious cause was diagnosed by appropriate microbiological tests. Different criteria were used for the diagnosis of: ocular tuberculosis (Gupta’s criteria) [21], spondyloarthritis (SpA) (ASAS criteria) [22], Behçet’s disease (International Study Group criteria) [23], Vogt–Koyanagi–Harada (VKH) disease (revised international committee criteria) [24], multiple sclerosis (MS) (revised McDonald’s criteria) [25], sarcoidosis (international criteria for the diagnosis of sarcoidosis or modified Abad’s criteria in the absence of histologic evidence) [5, 26], Crohn’s disease [27] and granulomatosis with polyangiitis [28]. For other causes, we used the SUN classification criteria developed for the 25 most frequent uveitis etiologies [29]. In the absence of classification criteria available, the etiological diagnosis was made by a consensus of two uveitis experts with a median follow-up of 28 months (range, 0–201) for idiopathic uveitis.

### Study factors

We selected 109 relevant demographic, ophthalmological, clinical, and biological factors (Table 1). Demographic factors were included: age at uveitis onset, ethnicity (Caucasians, North Africans, sub-Saharan Africans, Asians, and others), and gender. At the initial visit, classical ophthalmological characteristics according to SUN criteria (anatomical type of uveitis, chronicity, and laterality) and more specific associated signs were considered [29]. Data concerning anamnesis and extra-ophthalmological clinical examination were also included as well as paraclinical examinations recommended for all cases of uveitis. Due to our systematic approach to diagnosing uveitis using the ULISSE screening protocol in our tertiary center, only 12 out of 1249 patients had incomplete data.

**Table 1:** Study factors and etiologies included in the algorithm.

Group	Study factors
Demographic	Age at uveitis onset—Ethnicity—Gender
Ophthalmological semiology	
Anatomic	Anterior, Intermediate, Posterior, Panuveitis or Combined uveitis
Clinical course	Acute, Chronic, Relapse
Laterality	Unilateral or Bilateral
Associated specific signs	Anterior segment: granulomatous, ocular hypertension (>21 mmHg), synechiae, hypopyon, iris nodules (Koeppe or Busacca), iris heterochromia, stellated keratic precipitates, cataract, scleritis, episcleritis. Intermediate segment: vitritis, snowballs, snowbank. Posterior segment: focal or multifocal choroiditis, chorioretinal scars, chorioretinal node, choroidal white spots, serpiginous choroiditis, cystoid macular edema (by OCT), papillitis, vasculitis: venous or arterial/segmental or diffuse/occlusive or not (by fluorescein angiography), Capillaropathy, microaneurysms, neo-vessels, serous retinal detachment, retinitis, retinal necrosis
Extra-ophthalmological semiology	
Anamnesis	Trauma or eye surgery Drug-taking history Exposition to endemic disease Contact with animals (cat or dog) Forest walk
Physical examination	Joint: axial and peripheral joint pains, chondritis Skin: oral or bipolar aphthosis, pseudofolliculitis, psoriasis, poliosis/vitiligo, alopecia, early onset canities, skin rash, lupus pernio, sarcoid skin, infiltration of scar, erythema nodosum, zona Neurological: headaches, sensory and/or motor deficiency, facial nerve palsy, optic neuritis Pulmonary: dry cough, dyspnea Ear, Nose and Throat: sialadenitis, sialadenomegaly, hearing loss or tinnitus, crusty rhinitis. Abdominal: diarrhea, abdominal pain, splenomegaly, hepatomegaly Urological: epididymitis Lymph node: peripheral lymph node Vascular: venous or arterial thrombosis
Complementary examinations	Syphilis serology Interferon Gamma Release Assay (IGRA) or Tuberculin Skin Test (TST) Pulmonary X-ray Cell blood count (CBC)
Etiologies	Inflammatory diseases: sarcoidosis, ankylosing spondyloarthritis, HLA-B27 related uveitis, Behçet’s disease, Vogt–Koyanagi–Harada disease, multiple sclerosis, psoriatic arthritis, inflammatory bowel disease, juvenile idiopathic arthritis, Tubulo-interstitial nephritis and uveitis (TINU). Infection diseases: ocular tuberculosis, herpes: HSV/VZV, toxoplasmosis, syphilis, Lyme disease, toxocarosis. Pure-related ophthalmological diseases: Birdshot retinochoroidopathy, pars planitis, multifocal choroiditis with panuveitis, Fuchs disease, Posner–Schlossman syndrome. Others: idiopathic, primary Vitreoretinal lymphoma, drug-induced uveitis.

### Selected uveitis etiologies

All the most common causes (>0.5%) reported in the most recent European series were included [4]. We excluded etiologies whose number was less than three in the dataset. In the end, 25 causes of uveitis were included in the study: inflammatory and infectious diseases, pure ophthalmological entities, drug-induced uveitis, masquerade syndrome, and idiopathic uveitis (Tables 1 and 2). Uveitis was considered idiopathic after a comprehensive etiological workup and an unknown etiology after 1 year’s follow-up.

**Table 2:** Etiologies included in the algorithm analysis.

Group	Etiologies	Prevalence (*n*, %)
Inflammatory diseases	Sarcoidosis	245, 19.6%
	Ankylosing spondyloarthritis	59, 4.7%
	HLA-B27 related uveitis	53, 4.2%
	Behçet’s disease	40, 3.2%
	Vogt–Koyanagi–Harada disease	21, 1.7%
	Multiple sclerosis	21, 1.7%
	Psoriatic arthritis	10, 0.8%
	Inflammatory bowel disease	8, 0.6%
	Juvenile idiopathic arthritis	6, 0.48%
	Tubulo-interstitial nephritis and uveitis (TINU)	4, 0.3%
Infectious diseases	Ocular tuberculosis	84, 6.7%
	Herpes: HSV	20, 1.6%
	Toxoplasmosis	13, 1%
	Syphilis	12, 0.9%
	Lyme disease	11, 0.9%
	Toxocarosis	4, 0.3%
	Herpes: VZV	3, 0.2%
Pure-related ophthalmological diseases	Birdshot retinochoroidopathy	43, 3.4%
	Pars planitis	31, 2.5%
	Multifocal choroiditis with panuveitis	17, 1.4%
	Fuchs disease	14, 1.1%
	Posner–Schlossman syndrome	12, 1%
Others	Idiopathic	483, 38.7%
	Primary Vitreoretinal lymphoma	21, 1.7%
	Drug-induced uveitis	14, 1.1%

### Algorithm development

The neural network is developed to formulate a plausible hypothesis regarding the etiology of uveitis in patients by leveraging valuable information from clinical data. This model must have high accuracy in top-N etiologies prediction to prevent the model from excluding the real etiology.

#### Model selection and design

Artificial Neural Networks (ANN), specifically the Multilayer Perceptron (MLP), a feed-forward ANN with three layers have demonstrated the ability to automatically identify potential diseases based on patient symptoms and test results [30, 31]. MLP has been successfully applied in various fields, including image identification, and medical diagnosis [32, 33]. To evaluate the overall accuracy of Top-1 and Top-2 results, we used the decision tree as the benchmark for comparative analysis and also included two commonly used machine learning models, Support Vector Machine (SVM), and Random Forest (RF). The comparative analysis demonstrates good results for SVM (Top-1: 72.6%) and RF (Top-1: 76.3%). However, ANN-type MLP outperforms them in terms of Top-1 and Top-2 overall accuracy. Additionally, we observed that the decision tree’s performance is poor (Top-1: 68.0%) due to its susceptibility to overfitting when the training data are small, and it is sensitive to noise or small changes in the data (Table 3).

**Table 3:** Results of Support Vector Machine and Random Forest compared to Multilayer Perceptron.

	Decision Tree	Support Vector Machine	Random Forest	Multilayer Perceptron
Top-1	68.0 (67.6; 68.0)	72.6 (72.3; 73.0)	76.3 (75.9; 77.0)	**77.8 (77.4; 78.0)**
Top-2	68.3 (67.9; 69.0)	91.42 (91.2; 92.0)	92.3 (92.1; 93.0)	**93.0 (92.8; 93.1)**

Bold: performance of selected algorithm.

**Fig. 2:**
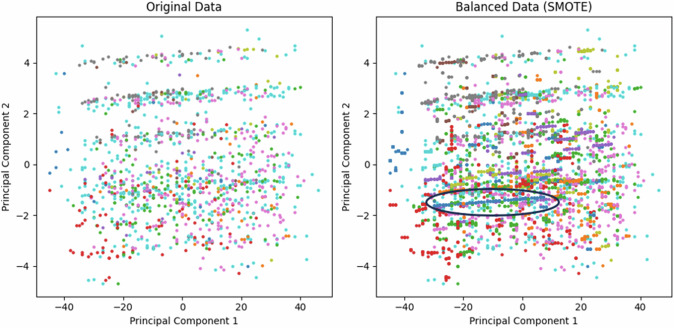
PCA dimensionality reduction figures of original data and data after SMOTE.

On the other hand, our data are extremely out of balance (Table 1). We employed the classic oversampling algorithm Synthetic Minority Oversampling Technique (SMOTE) to perform an experimental analysis, i.e., to balance the dataset by adding minority class samples [34]. According to the experiment’s results, there was an 8.8% decrease in etiology diagnosis. To analyze the reasons for the performance degradation, we use principal component analysis (PCA) to reduce the data to two dimensions for visualization (Fig. 2). We can observe that after using the oversampling method, the etiology distribution has changed significantly. For example, the classification in the black circle presents a different distribution from the original data after oversampling. Therefore, it is risky to use some oversampling algorithms to balance the data for the diagnosis of uveitis etiology. Because it easily causes the dataset to lose its real distribution characteristics and information, creates some false cases, and affects the accuracy and comprehensive diagnostic analysis of the cause.

#### Neural network type MLP

**Fig. 3:**
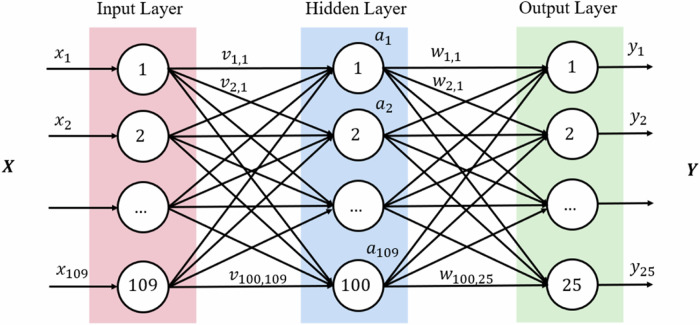
The structure of the designed neural network.

The structure of the designed neural network is shown in Fig. 3. In this network, the central node represents a neuron. The leftmost layer is called the input layer and consists of a set of neurons with the same number of neurons as the study factors. Followed by the hidden layer, each neuron in the hidden transforms the value of the previous layer by weighted linear summation, which enables the MLP to model data with complex nonlinear relationships. Finally, the output layer of the MLP converts the output into a probability distribution ***Y*** = *y*_1_, *y*_2_, …, *y*_25_ for each etiology using a softmax activation function. Since each output neuron is associated with a specific etiology, the number of neurons in the output layer is equal to the number of etiology. When training the model, we input the patient’s study factors such as age, gender, etiology-related factors ***X*** = *x*_1_, *x*_2_,…, *x*_109_, and the corresponding etiology *y* into the network. We optimized the MLP algorithm’s parameters using the Bayesian optimization algorithm to improve the diagnostic performance of the adopted MLP algorithm [35]. Table 4 displays the parameter space that was used as well as the parameters that were ultimately chosen. After the model has been trained, the study factors of the patients, who were not included in the training, are introduced to the network as input data, and the network then automatically outputs the probability for each etiology.

**Table 4:** The parameter space.

Parameter name	Parameter range	Selected values
hidden_layer_sizes	Integer, range: (100, 500, 100)	200
Alpha	Floating, range: (0.0001, 0.1)	0.0001
Activation	List, optional values: ['relu', 'tanh', 'logistic']	'relu'
Optimizer	List, optional values: ['adam', 'sgd', 'lbfgs']	'adam'
learning_rate	Floating, range: (0.0001, 0.1)	0.001

Since our study included *n* (*n* = 25) etiologies and it is a multi-classification problem, we first need to define the confusion matrix of multi-etiologies diagnosis results. According to the confusion matrix, we can calculate the value of the accuracy, which is the most commonly used indicator in multi-classification problems. As it can calculate the ratio of the number of correct classification predictions to the total number of predictions, we employ Accuracy as the main evaluation indicator to measure performance. In detail, the Accuracy can be computed by

$${Accuracy}=\frac{{\sum }_{i=1}^{n}{T}_{{ii}}}{N}$$

where *N* is the total number of data, and *T*_*ii*_ is the number of successfully predicting the *i*th etiology.

We assume that the dataset is *D* = {*X,Y*}. The training and evaluation steps of the proposed MLP model are as follows:

- Divide the dataset into training set *D*_*training*_, verification set, and test set *D*_*testing*_;
- Use the training set *D*_*training*_ to train the MLP model;
- The trained MLP model is used to output the probabilities of *n* patients in the validation set *D*_*validation*_, which are expressed as ${P}_{m}=({P}_{m1},{P}_{m2},\ldots {P}_{{mn}})$.
- Use the validation set to search for the optimal parameter N of the MLP model in the parameter space *N* = *range*(1,10,1);
- Finally, the test set *D*_*testing*_ is used to evaluate the PFPM model’s performance.

It is worth noting that in the actual experiment, we sampled 100 training and test cases on the data, and the final result displayed is the average of 100 results. This can guarantee the repeatability of the results and lessen unintentional errors brought on by the randomness of a single sampling result. Concurrently, we implemented the “StratifiedShuffleSplit” cross-validation technique for dataset splitting, which guarantees that the training and test sets’ category distributions resemble the original dataset, thereby minimizing model deviation.

### Ethics statement

According to French law (no. 2004-806, 9 August 2004), and because the data were collected retrospectively and patient management was not modified, this study did not require research ethics committee approval. However, the cohort study received approval from the local ethics committee (Hospices Civils de Lyon) in February 2019 (No. 19-31) and was registered on clinicaltrials.gov (NCT 03863782).

## Results

### Description of the study populations

One thousand two hundred forty-nine patients received an uveitis diagnosis in Croix-Rousse University Hospital between January 2003 and December 2021. Among these, 874 patients (70%) were randomly selected for the training dataset and 375 (30%) for the test dataset.

In these populations, the median [IQR] age was 47 [31;63] years, and 738 patients (59.1%) were women. Caucasians represented the most frequent ethnicity (79.2%), followed by North Africans (17.7%). Regarding ophthalmological characteristics, the most frequent anatomic type was anterior uveitis with 408 patients (32.7%) followed by panuveitis with 366 patients (29.3%). Uveitis was chronic in 68.2% of the cases and unilateral in 40.4%. Concerning etiology, uveitis was idiopathic in 38.7% of the patients and sarcoidosis represented the first identified etiology (19.6%) followed by ocular tuberculosis (6.7%).

### Diagnostic performance metrics

The algorithm’s most probable diagnosis (Top-1) matched the senior clinician diagnosis in 292 of 375 patients (77.8%, 95% CI: 77.4–78.0). It reached 93% (95% CI: 92.8–93.1%) when the two most probable diagnoses (Top-2) were considered among the 375 patients of the test dataset.

Table 5 shows the sensitivities, specificities, and positive predictive value estimates of the algorithm for each individual diagnosis. Considering only the algorithm’s Top-1, sensitivity estimates for each etiology ranged from 0% (patients with TINU syndrome) to 97% (patients with syphilitic uveitis). When the Top-2 were considered, the sensitivity estimates increased from 0% to 100%, respectively. Mean sensitivity estimates for the Top-1 were over 70% for more than three-quarters of etiologies (19/25). Considering only the Top-2, specificity estimates for each etiology ranged from 82% (patients with idiopathic uveitis) to 100% (18 etiologies/25). The algorithm performed well in diagnosing idiopathic uveitis, with a sensitivity estimate of 81% and a specificity estimate of 82%. Difficult-to-diagnose etiologies of uveitis, such as systemic diseases, are mostly well-diagnosed by the algorithm with a mean sensitivity estimate of 78%, a mean specificity estimate of 99%, and a mean positive predictive value estimate of 84%. Thanks to a large number of patients for most etiologies, estimates of performance metrics can be reliably interpreted.

**Table 5:** Sensitivity, specificity, and positive predictive value of the Neural network algorithm to identify each uveitis etiology in the test dataset.

Etiology	Most probable diagnosis			Two most probable diagnoses		
	Sensitivity (95% CI)	Specificity (95% CI)	Positive predictive value (95% CI)	Sensitivity (95% CI)	Specificity (95% CI)	Positive predictive value (95% CI)
Idiopathic	0.81 (0.77; 0.85)	0.82 (0.81; 0.83)	0.74 (0.74; 0.75)	0.96 (0.95; 0.96)	0.44 (0.43; 0.45)	0.52 (0.52; 0.52)
Sarcoidosis	0.70 (0.69; 0.71)	0.94 (0.94; 0.95)	0.76 (0.75; 0.77)	0.92 (0.91; 0.92)	0.63 (0.63; 0.64)	0.38 (0.38; 0.38)
Tuberculosis	0.92 (0.91; 0.93)	1 (1; 1)	0.94 (0.93; 0.95)	0.98 (0.97; 0.98)	0.96 (0.96; 0.97)	0.66 (0.64; 0.68)
Ankylosing SpA	0.90 (0.89; 0.92)	0.99 (0.99; 0.99)	0.87 (0.86; 0.88)	0.97 (0.96; 0.98)	0.98 (0.98; 0.98)	0.74 (0.72; 0.75)
HLA-B27 related uveitis	0.44 (0.41; 0.46)	0.98 (0.98; 0.98)	0.52 (0.50; 0.55)	0.79 (0.78; 0.81)	0.90 (0.90; 0.90)	0.27 (0.26; 0.27)
Birdshot disease	0.91 (0.90; 0.93)	1 (1; 1)	0.90 (0.89; 0.91)	0.95 (0.94; 0.97)	0.97 (0.97; 0.98)	0.58 (0.56; 0.59)
Behçet’s disease	0.90 (0.89; 0.93)	1 (1; 1)	0.95 (0.93; 0.96)	0.98 (0.97; 0.98)	1 (1; 1)	0.62 (0.60; 0.64)
Pars planitis	0.81 (0.78; 0.84)	1 (0.99; 1)	0.82 (0.80; 0.85)	0.98 (0.97; 0.99)	0.98 (0.97; 0.98)	0.52 (0.50; 0.54)
VKH disease	0.81 (0.78; 0.84)	1 (1; 1)	0.91 (0.89; 0.93)	0.93 (0.91; 0.95)	0.99 (0.99; 0.99)	0.64 (0.62; 0.67)
Multiple sclerosis	0.79 (0.76; 0.83)	0.99 (0.99; 1)	0.77 (0.74; 0.80)	0.94 (0.92; 0.95)	0.98 (0.98; 0.98)	0.52 (0.50; 0.54)
Vitreoretinal lymphoma	0.50 (0.46; 0.54)	0.99 (0.99; 1)	0.64 (0.59; 0.68)	0.77 (0.74; 0.80)	0.95 (0.95; 0.95)	0.21 (0.20; 0.22)
Herpes Simplex Virus	0.37 (0.34; 0.40)	0.99 (0.99; 0.99)	0.47 (0.43; 0.51)	0.60 (0.57; 0.64)	0.96 (0.95; 0.96)	0.18 (0.17; 0.19)
Multifocal choroiditis	0.94 (0.92; 0.96)	1 (1; 1)	0.98 (0.97; 0.99)	0.96 (0.95; 0.98)	1 (0.99; 1)	0.76 (0.73; 0.78)
Fuchs disease	0.90 (0.88; 0.93)	1 (1; 1)	0.93 (0.91; 0.95)	0.93 (0.91; 0.95)	1 (1; 1)	0.87 (0.85; 0.90)
Drug-induced uveitis	0.81 (0.77; 0.85)	1 (1; 1)	0.98 (0.97; 0.99)	0.85 (0.81; 0.88)	0.99 (0.99; 0.99)	0.48 (0.44; 0.52)
Toxoplasmosis	0.78 (0.73; 0.82)	1 (1; 1)	0.85 (0.81; 0.88)	0.92 (0.89; 0.94)	0.98 (0.98; 0.98)	0.35 (0.33; 0.38)
Posner–Schlossman	0.83 (0.79; 0.87)	1 (1; 1)	0.82 (0.78; 0.86)	0.90 (0.87; 0.93)	0.99 (0.99; 0.99)	0.65 (0.62; 0.69)
Syphilis	0.97 (0.96; 0.99)	1 (1; 1)	1 (1; 1)	1 (1; 1)	1 (1; 1)	0.89 (0.87; 0.91)
Lyme disease	0.99 (0.98; 1)	1 (1; 1)	0.99 (0.98; 1)	1 (1; 1)	0.99 (0.99; 0.99)	0.61 (0.58; 0.65)
Psoriatic arthritis	0.73 (0.67; 0.78)	1 (1; 1)	0.77 (0.72; 0.82)	0.99 (0.98; 1)	0.99 (0.99; 0.99)	0.53 (0.50; 0.56)
IBD	0.87 (0.77; 0.85)	1 (1; 1)	0.97 (0.95; 0.99)	0.89 (0.85; 0.93)	0.99 (0.99; 1)	0.87 (0.83; 0.90)
JIA	0.54 (0.47; 0.60)	1 (1; 1)	0.75 (0.67; 0.83)	0.71 (0.65; 0.77)	0.99 (0.99; 0.99)	0.27 (0.23; 0.30)
TINU syndrome	0.0 (0.0; 0.0)	1 (1; 1)	0.0 (0.0; 0.0)	0.0 (0.0; 0.0)	0.99 (0.98; 0.99)	0.0 (0.0; 0.0)
Toxocariasis	0.88 (0.82; 0.94)	1 (1; 1)	0.88 (0.82; 0.94)	0.91 (0.85; 0.97)	1 (1; 1)	0.89 (0.83; 0.95)
Varicella-zoster virus	0.50 (0.40; 0.60)	1 (1; 1)	0.50 (0.40; 0.60)	0.78 (0.70; 0.86)	1 (1; 1)	0.41 (0.35; 0.48)

*IBD* inflammatory bowel disease, *JIA* juvenile idiopathic arthritis.

### Algorithm mispredictions

**Fig. 4:**
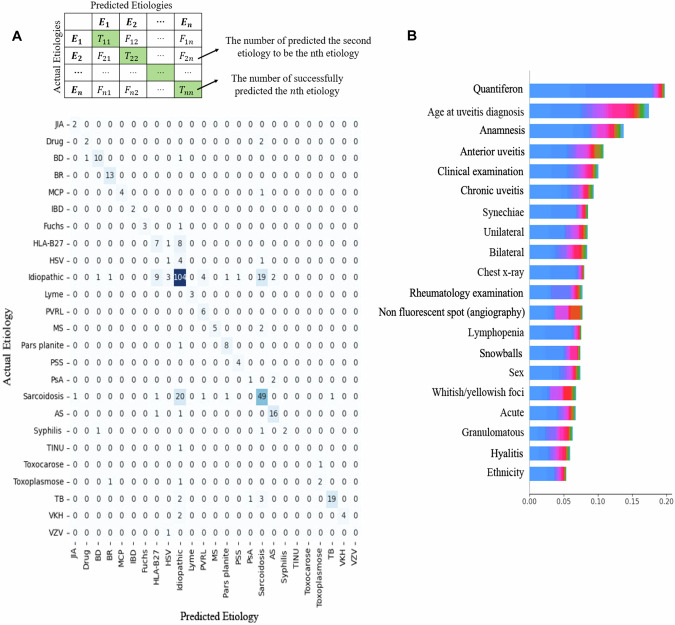
Etiological classification matrix. **A** Confusion matrix of multi-etiologies diagnosis confirmed by experts (actual) versus those predicted by the algorithm (predicted). **B** The relative weights and impact of features on the prediction outcomes.

Some etiologies were most commonly confused by the algorithm (Fig. 4A). According to the confusion matrix, we can observe that the model easily mispredicts idiopathic uveitis as ocular sarcoidosis (*n* = 5) or vitreoretinal lymphoma (*n* = 2). Ocular sarcoidosis and HLA-B27-related uveitis are also frequently mispredicted as idiopathic uveitis in 20 and 6 cases respectively. Other mispredictions observed were not repetitive.

Concerning etiologies with poor sensitivity, HLA-B27-related uveitis, ocular sarcoidosis, and primary vitreoretinal lymphoma were frequently confused with idiopathic uveitis. In case of misprediction of these two etiologies as idiopathic uveitis, Top-2 usually included the correct etiology, with sensitivity increasing to 79% (vs 44% for Top-1) for HLA-B27-related uveitis, 92% (vs 70% for Top-1) for ocular sarcoidosis and 77% (vs 50% for Top-1) for primary vitreoretinal lymphoma. Identifying these etiologies can be challenging due to the lack of specific ocular signs. Additional examinations are necessary for an accurate etiological diagnosis. For HSV, VZV, TINU syndrome, and juvenile idiopathic arthritis, there are not enough cases in the database to give a reliable interpretation of the prediction errors.

### Discriminating factors

A graphical tool for evaluating machine learning models called SHAP (SHapley Additive exPlanations) was utilized to assess which factors in the algorithm are most discriminative in determining the etiology of uveitis [36]. This tool can display the relative weights of characteristics inside the model and their impact on the prediction outcomes (Fig. 4B).

Globally, the most important feature was the “Quantiferon”, especially to predict the diagnosis of idiopathic uveitis which represented the most important etiology in our database. All demographic factors had a noteworthy influence on the prediction of the etiology, with “Age” being particularly significant. Some of the discriminating ophthalmologic characteristics were “Anterior uveitis”, “Chronic uveitis”, “Synechiae”, “Laterality”, and “Granulomatous”. “Anamnesis” and “Clinical examination” were also relevant for prediction, particularly “Rheumatology examination” for diagnosing SpA-related-uveitis.

For individual prediction of each etiology, the main discriminating feature was ophthalmological in most cases: “Non-granulomatous” for juvenile idiopathic arthritis; “White foci on posterior segment” for Birdshot Disease, primary vitreoretinal lymphoma, toxocariasis, and toxoplasmosis; “Iritis heterochromia” for Fuchs Disease; “Recurrence” for HLA-B27-related disease; “Unilateral” for HSV; “Non-posterior uveitis” for Posner–Schlossman syndrome; “Hyalitis” for Sarcoidosis; “Granulomatous” for Syphilis; “Posterior uveitis” for ocular tuberculosis; “Serous Retinal detachment” for VKH disease. Anamnesis and clinical examination were also the main discriminating feature in some cases: “Anamnesis” for drug-related uveitis, Lyme disease, and Psoriatic arthritis; “oral aphthosis” for Behçet disease; “Axial joint pain” for ankylosing spondylitis; “contributory neurological examination” for MS; “normal clinical examination” for pars planitis and TINU syndrome.

## Discussion

### Principal results

This Neural network (MLP) enables the identification of uveitis etiology with high performance when seeking the most likely diagnoses. The results from the test dataset revealed that the etiology of uveitis, as determined by experts corresponded to the most probable diagnosis in at least 78% of cases. A key strength of our diagnostic decision support system (DDSS) lies in its good diagnostic performance, with sensitivity exceeding 70% and specificity surpassing 80% for more than three-quarters of etiologies, particularly for complex and/or rare cases. Similar to previous DDSS, our algorithm exhibits high specificities, allowing the exclusion of less likely etiologies. However, sensitivities for each etiology vary significantly. Some etiologies are responsible for uveitis without very specific ocular or extraocular signs (e.g., HLA-B27-related uveitis, herpetic uveitis) and are misclassified as idiopathic in our algorithm’s Top-1. Fortunately, our algorithm’s Top-2 proposals often include these etiologies and are more useful for clinicians to consider. This helps ensure that a misclassified but frequent etiology is not overlooked.

Our algorithm mainly used classic ophthalmological features to predict uveitis etiology such as anatomical description, evolution, and laterality. Associated signs (granulomatous, synechiae, and white foci on the posterior segment) were also relevant. However, anamnesis, demographical, and clinical features were discriminant, particularly for systemic diseases and infectious diseases related to uveitis. Interestingly, the algorithm’s discriminating features correspond to the features that experts believe should guide the etiological workup [4].

### Comparison with prior work

Expert clinicians use the algorithmic approach to assess the underlying etiology of uveitis [4]. This approach starts with history taking and demographics, followed by consideration of ocular examination characteristics, and finally the assessment of physical signs of extraocular disease. For years, experts have agreed on minimal paraclinical investigations required for all types of uveitis such as syphilis serology, chest X-ray, Interferon-Gamma Release Assays (IGRA), and Cell Blood Count (CBC) [8].

DDSSs have been developed to assist in uveitis diagnosis [13, 14, 16, 18] (Table 6). Previous studies found that the etiology determined by the senior clinician matched the first probable diagnosis given by an algorithm from 54% to 74% of cases. Our algorithm showed superior performance with a match for the most probable diagnosis in 78% of cases. Compared to existing DDSS, our algorithm addresses the limitations hindering their clinical practice implementation [13, 14, 16, 18]. Notably, some knowledge bases include diagnoses with very high specificity tests and/or ophthalmological entities easily recognized by the ophthalmologist leading to an overestimation of diagnostic performance. However, the primary challenge faced by specialists is determining rare or difficult diagnoses to avoid leaving too many patients with a diagnosis of idiopathic uveitis. Our DDSS is specifically designed to tackle this daily issue. Moreover, certain anatomical types of uveitis (focus on anterior uveitis) and diagnostically complex etiologies are not included in previous algorithms limiting their utility to cases of diagnostic uncertainty [13, 14]. In contrast, our algorithm is suitable for all uveitis with a complex etiological diagnosis. Furthermore, response to treatment (corticosteroids response) is considered in two DDSS, but not included in our algorithm [16, 18]. While incorporating this study factor could improve diagnostic performances, it will delay the use of the DDSS until ocular diagnosis is made. Our study is the first to incorporate new demographic elements (e.g., ethnicity), additional ophthalmic variables (e.g., presence of papilledema or focal or diffuse choroiditis), and detailed clinical examination findings on a larger number of cases, potentially enhancing our results.

**Table 6:** Comparison of our algorithm with the previous algorithm.

Study	Methods	Study factors	Etiology	Diagnostic performance
González-López et al. [14]	Bayesian belief networks Data set: 200 cases (anterior uveitis)	Demographic (gender) Ophthalmic (ocular symptoms and signs) Clinical examination (systemic symptoms and signs) Laboratory tests	11	Sensitivity (most probable etiology): 64% Sensitivity (two most probable etiologies): 81%
Gegundez-Fernandez et al. [18] (Uvemaster)	Interference method with filtering rules Data set: 88 cases (all uveitis type)	Demographic (age, gender, immunodeficiency, drugs, trauma or eye surgery, endemic disease) Ophthalmic (anatomy, chronicity, laterality, granulomatous, vasculitis, papillitis, scleritis, specific ocular involvement) Clinical examination (skin, mucosal, nervous system, articular, urinary, ear, nose, throat, digestive and cardiovascular exam) Treatment (steroid response)	88	Sensitivity (most probable etiology): 74% Sensitivity (three most probable etiologies): 91%
Mutawa et al. [16]	Rule-based expert system (multilayer rule design) Data set: 61 cases (case report, all uveitis type)	Ophthalmic (anatomy, chronicity, severity, laterality, granulomatous) Treatment (response to therapy)	53	Sensitivity (most probable etiology): 60% Sensitivity (four most probable etiologies): 100%
Jamilloux et al. [13]	Bayesian belief networks Data set: 877 cases (all uveitis type)	Demographic (Age, sex, ethnicity) Ophthalmic (Anatomy, laterality, chronicity, vasculitis, granulomatous, ocular hypertension)	8	Sensitivity (most probable etiology): 54% Sensitivity (two most probable etiologies): 85%
Our algorithm	Neural Network Multi-layered perceptron Data set: 1249 cases (all uveitis type)	Demmographic (age, sex, ethnicity) Ophtalmic (Anatomy, chronicity, laterality, granulomatous, vasculitis, papillitis, synechiae, specific ocular involvement) Clinical examination (skin, nervous system, articular, urinary, ear, nose, throat, digestive, lung and cardiovascular exam Complementary exams (Syphilis serology, IGRA or TST, Pulmonary X-ray, CBC)	25	Sensitivity (most probable etiology): 77.8%. Sensitivity (two most probable etiologies): 93%.

### Interpretability and clinical integration

**Fig. 5:**
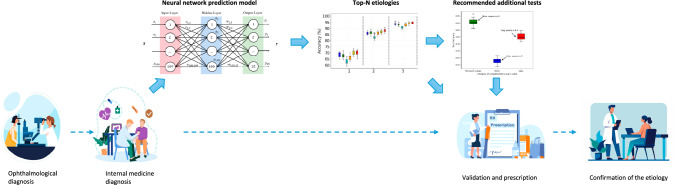
Medical decision-making tool for uveitis clinicians.

The clinician aims to assess fastly uveitis etiology with minimal complementary examinations and invasive tests. This algorithm was designed as a medical decision-making tool, highlighting probable etiologies, to aid in selecting further investigations for non-expert uveitis clinicians (Fig. 5). We excluded etiologies that are easily diagnosed by the ophthalmologist (pure-related-ophthalmological disorder) to intentionally address challenging diagnoses.

With an accuracy of 93% considering the Top-2 predicting with our algorithm, it seems worthwhile to firstly conduct the complementary exams first, to diagnose the etiologies included in this Top-2. This would enable a quick assessment of etiological diagnosis, and better targeting of essential exams, all while limiting costs and avoiding invasive and irradiating procedures. In addition, our DDSS performs well in identifying idiopathic uveitis, where the etiological workup should be probably limited. For etiologies with sensitivity <90% according to Top-2 estimates (e.g, HLA-B27-related disease, primary vitreoretinal lymphoma, HSV, VZV, juvenile idiopathic arthritis, and TINU syndrome), we recommend considering these potential causes during an etiological workup and remaining cautious throughout the process.

Thanks to the observation of discriminating features the algorithm also assists clinicians in selecting the probable etiology. This allows clinicians to identify relevant features for suspected causes.

In the future, this DDSS should undergo validation on an international cohort and be evaluated in a prospective study to compare its diagnostic performance. A diagnostic tool is under development to interface with electronic medical records for prospective evaluation. Ease of implementation will be assessed with clinicians. It has the potential to aid clinicians in the case of undetermined uveitis after an initial ophthalmological examination. However, an accurate description of ophthalmological and extra-ophthalmological examination is still necessary to maximize performance. Nonetheless, clinicians continue to play a crucial role in assessing the underlying etiology of uveitis.

### Limitations

Our study has a series of limitations. Firstly, its retrospective nature and some ophthalmological and extra-ophthalmological features may have been omitted in the consultation reports which could not be entered in the DDSS. However, our tertiary centers have implemented a systematic approach to the etiological diagnosis of uveitis, ensuring minimal missing data. Secondly, patient recruitment at a tertiary internal medicine center may have resulted in an increased frequency of underlying multisystemic diseases and the inclusion of more severe cases which may lead to difficulties of implementation to other populations. Additionally, the study was conducted in France, not an endemic area with the majority of the ethnic group being Caucasian making the results non-extrapolatable to other countries. Moreover, specific etiologies in the test dataset have a small sample size (e.g., TINU syndrome, HSV, and VZV) which affects the reliability of diagnostic performance indicators for these cases. Lastly, our algorithm has not yet been validated on an independent cohort.

## Conclusion

Our study represents an accurate algorithm for the clinical management of uveitis with undetermined etiology after an ophthalmological examination. The diagnoses generated by the algorithm can be considered as an aid for the etiological assessment. Its performance needs to be confirmed in larger and prospective studies, such as cluster randomized studies.

## Summary

### What was known before

- Evaluation of four diagnosis decision support systems in uveitis found a correct diagnosis from 54% to 74% of cases.
- These computer-aided systems have limitations and are not used in daily clinical practice.

### What this study adds

- The most probable diagnosis of uveitis with unknown etiology by the neural network matched the senior clinician diagnosis in 292 of 375 patients (77.8%). It reached 93% when the two most probable diagnoses were considered.
- This algorithm might be used as a medical decision-making tool for the clinical management of uveitis with undetermined etiology after an ophthalmological examination.

## Supplementary information

Figures 1, 2, 3 and Tables 1, 2, 3, 4

## Data Availability

The data used during this study are available from the corresponding author on reasonable request.
