# Supplementary material for: Neural networks for predicting etiological diagnosis of uveitis
Source: Eye (Lond). 2024 Dec 20;39(5):992–1002. doi: 10.1038/s41433-024-03530-2 (PMC11933267; doi:10.1038/s41433-024-03530-2)
Supplement: Supplementary file 1 — Figures 1, 2, 3 and Tables 1, 2, 3, 4 [file 41433_2024_3530_MOESM1_ESM.docx]

| **Group** | **Etiologies** | **Prevalence (n, %)** |
| --- | --- | --- |
| Inflammatory diseases | Sarcoidosis  Ankylosing spondyloarthritis  HLA-B27 related uveitis  Behçet’s disease  Vogt-Koyanagi-Harada disease  Multiple sclerosis  Psoriatic arthritis  Inflammatory bowel disease  Juvenile idiopathic arthritis  Tubulo-interstitial nephritis and uveitis (TINU) | 245, 19.6%  59, 4.7%  53, 4.2%  40, 3.2%  21, 1.7%  21, 1.7%  10, 0.8%  8, 0.6%  6, 0.48%  4, 0.3% |
| Infectious diseases | Ocular tuberculosis  Herpes: HSV  Toxoplasmosis  Syphilis  Lyme disease  Toxocarosis  Herpes: VZV | 84, 6.7%  20, 1.6%  13, 1%  12, 0.9%  11, 0.9%  4, 0.3%  3, 0.2% |
| Pure related ophthalmological diseases | Birdshot retinochoroidopathy  Pars planitis  Multifocal choroiditis with panuveitis  Fuchs disease  Posner-Schlossman syndrome | 43, 3.4%  31, 2.5%  17, 1.4%  14, 1.1%  12, 1% |
| Others | Idiopathic  Primary Vitreoretinal lymphoma  Drug induced uveitis | 483, 38.7%  21, 1.7%  14, 1.1% |

**Table 2.** Etiologies included in the algorithm analysis.

|  | **Decision Tree** | **Support Vector Machine** | **Random Forest** | **Multi-layer Perceptron** |
| --- | --- | --- | --- | --- |
| Top-1 | 68.0(67.6;68.0) | 72.6(72.3;73.0) | 76.3(75.9;77.0) | **77.8(77.4;78.0)** |
| Top-2 | 68.3(67.9;69.0) | 91.42(91.2;92.0) | 92.3(92.1;93.0) | **93.0 (92.8;93.1)** |

**Table 3**. Results of Support Vector Machine and Random Forest compared to Multi-layer Perceptron.

| Parameter Name | Parameter Range | Selected Values |
| --- | --- | --- |
| hidden_layer_sizes | Integer, range: (100, 500, 100) | 200 |
| Alpha | Floating, range: (0.0001, 0.1) | 0.0001 |
| Activation | List, optional values: ['relu', 'tanh', 'logistic'] | 'relu' |
| Optimizer | List, optional values: ['adam', 'sgd', 'lbfgs'] | 'adam' |
| learning_rate | Floating, range: (0.0001, 0.1) | 0.001 |

**Table 4.** The parameter space.

| Group | Study factors |
| --- | --- |
| Demographic | Age at uveitis onset – Ethnicity – Gender |
| Ophthalmological semiology  Anatomic  Clinical course  Laterality  Associated specific signs | Anterior, Intermediate, Posterior, Panuveitis or Combined uveitis  Acute, Chronic, Relapse  Unilateral or Bilateral  Anterior segment: granulomatous, ocular hypertension (> 21 mmHg), synechiae, hypopyon, iris nodules (Koeppe or Busacca), iris heterochromia, stellated keratic precipitates, cataract, scleritis, episcleritis.  Intermediate segment: vitritis, snowballs, snowbank.  Posterior segment: focal or multifocal choroiditis, chorioretinal scars, chorioretinal node, choroidal white spots, serpiginous choroiditis, cystoid macular oedema (by OCT), papillitis, vasculitis: venous or arterial/segmental or diffuse/occlusive or not (by fluorescein angiography), Capillaropathy, microaneurysms, neo-vessels, serous retinal detachment, retinitis, retinal necrosis |
| Extra-ophthalmological semiology  Anamnesis  Physical examination | Trauma or eye surgery  Drug-taking history  Exposition to endemic disease  Contact with animals (cat or dog)  Forest walk  Joint: axial and peripheral joint pains, chondritis  Skin: oral or bipolar aphtosis, pseudofolliculitis, psoriasis, poliosis/vitiligo, alopecia, early onset canitis, skin rash, lupus pernio, sarcoid skin, infiltration of scar, erythema nodosum, zona  Neurological: headaches, sensory and/or motor deficiency, facial nerve palsy, optic neuritis  Pulmonary: dry cough, dyspnea  Ear, Nose and Throat: Ssaladenitis, sialadenomegaly, hearing loss or tinnitus, crusty rhinitis.  Abdominal: diarrhea, abdominal pain, splenomegaly, hepatomegaly  Urological: epididymitis  Lymph node: peripheral lymph node  Vascular: venous or arterial thrombosis |
| Complementary examinations | Syphilis serology  Interferon Gamma Release Assay (IGRA) or Tuberculin Skin Test (TST)  Pulmonary X-ray  Cell blood count (CBC) |
| Etiologies | Inflammatory diseases: sarcoidosis, ankylosing spondyloarthritis, HLA-B27 related uveitis, Behçet’s disease, Vogt-Koyanagi-Harada disease, multiple sclerosis, psoriatic arthritis, inflammatory bowel disease, juvenile idiopathic arthritis, Tubulo-interstitial nephritis and uveitis (TINU).  Infection diseases: ocular tuberculosis, herpes: HSV/VZV, toxoplasmosis, syphilis, Lyme disease, toxocarosis.  Pure related ophthalmological diseases: Birdshot retinochoroidopathy, pars planitis, multifocal choroiditis with panuveitis, Fuchs disease, Posner-Schlossman syndrome.  Others: idiopathic, primary Vitreoretinal lymphoma, drug induced uveitis. |

**Table 1.** Study factors and etiologies included in the algorithm.


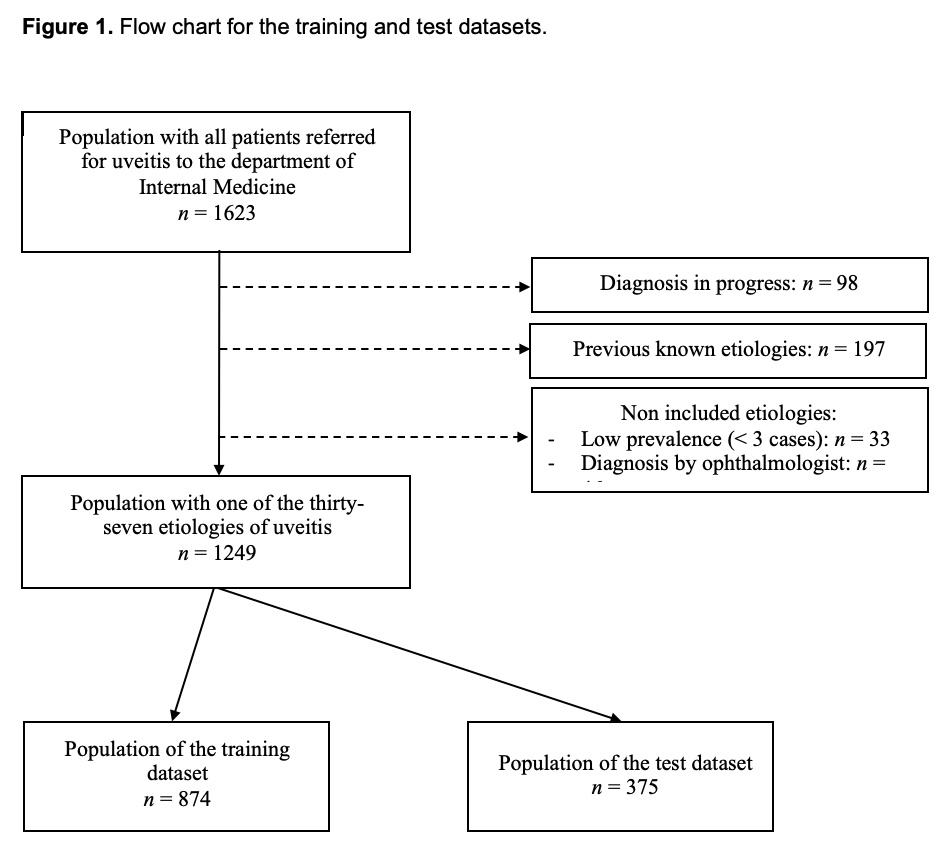


**Figure 1.** Flow chart for the training and test datasets


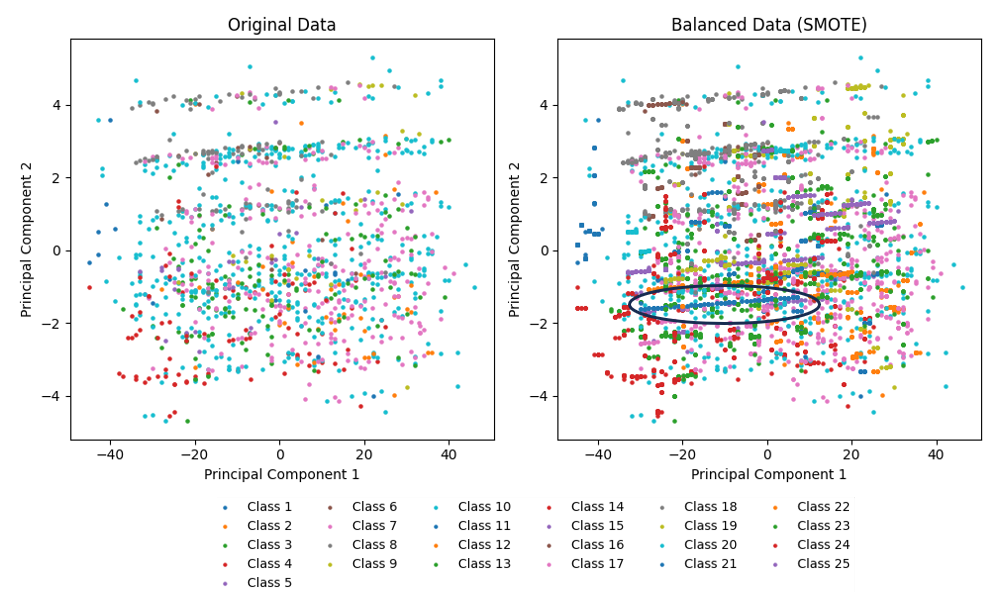


**Figure 2.** PCA dimensionality reduction figures of original data and data after SMOTE.
